# Deep Brain Stimulation Modulates Nucleus Accumbens Theta-Band Power in a Rodent Model of Schizophrenia

**DOI:** 10.3390/brainsci16101003

**Published:** 2026-09-22

**Authors:** Nadja Freund, Patrick R. Reinhardt, Mate Abraham, Klaus Funke, Georg Juckel

**Affiliations:** 1 Division of Experimental and Molecular Psychiatry, Department of Psychiatry, Psychotherapy and Preventive Medicine, LWL-University Hospital, Ruhr-University Bochum, D-44801 Bochum, Germany; nadja.freund@rub.de (N.F.);; 2 Department of Neurophysiology, Medical Faculty, Ruhr-University Bochum, D-44801 Bochum, Germany

**Keywords:** PolyI:C, electrophysiology, maternal immune activation, local field potentials, treatment

## Abstract

**Highlights:**

**What are the main findings?**

- In the maternal immune activation (MIA) model for schizophrenia, offspring showed overall reduced alpha- and beta-band power in the medial prefrontal cortex and lower ventral hippocampal firing rates at baseline.
- High-frequency mPFC DBS evoked a transient increase in spiking and bursting across the limbic system; within the first minute after stimulation, accumbal theta-band power was lower in MIA animals.

**What are the implications of the main findings?**

- The electrophysiological alterations in the model parallel abnormalities reported in schizophrenia, supporting the translational relevance and identifying mPFC alpha-/beta-band power and accumbal theta as possible candidate neurophysiological markers for intervention.
- The results motivate large-scale preclinical studies to guide clinical translation.

**Abstract:**

Background/Objectives: Deep brain stimulation has proven a powerful treatment option for various neuropsychiatric diseases. In psychiatry, this is particularly relevant for schizophrenia, where up to one third of patients do not respond to current treatment options. To further understand the possible mechanisms of deep brain stimulation for schizophrenia, this study combined deep brain stimulation with electrophysiological recordings. Methods: Injections of polyriboinosinic/polyribocytidilic acid were administered during pregnancy to induce a well-established maternal immune activation model of schizophrenia. Seven model offspring animals and nine control male Wistar rats underwent a high-frequency deep brain stimulation protocol. Electrophysiological recordings in the medial prefrontal cortex, the nucleus accumbens, the ventral hippocampus and the ventral tegmental area were performed before and after stimulation. Results: Maternal immune activation caused overall changes in local field potentials in the prefrontal cortex and neuronal spiking in the ventral hippocampus. In all recorded regions a transient effect of stimulation with increased spikes and bursts was visible. However, only in the nucleus accumbens was an effect following stimulation visible. Here, a significant group difference in theta-band power was seen directly after stimulation. Taken together, these results add evidence that stimulation of the prefrontal cortex may represent a possible method to modulate neuronal firing within the mesolimbic system in schizophrenia. Conclusions: To conclude, this study provides exploratory insights into the mechanisms of deep brain stimulation in a rodent model of schizophrenia. The results warrant further investigation in large-scale preclinical studies to shed light on the mechanisms of deep brain stimulation and ultimately inform future directions in psychiatric care.

## 1. Introduction

Deep brain stimulation (DBS) has experienced a surge in popularity over the last few decades [1]. Applications for neurological disorders such as Parkinson’s disease are well established and have developed into a second line of treatment [2].

Given this success, DBS is often discussed for treatment-resistant psychiatric disorders. However, so far, only a few approaches have shown convincing success, and large-scale clinical studies are still missing [3]. Especially for schizophrenia (SCZ), where up to one-third of patients are classified as treatment resistant, not responding to appropriate treatment with at least two different antipsychotics [4], there is an apparent need for new treatment options. While DBS holds strong potential for patients with treatment-resistant SCZ [5], so far, mainly only case studies and small pilot trials have been reported [6,7,8,9].

To further progress this line of research, preclinical data from animal studies can be used to inform future human studies. For SCZ one of the most well established models utilizes prenatal infection, a known risk factor for the development of SCZ [10], to induce a SCZ-like phenotype in rodents. Injections of the viral mimic polyriboinosinic/polyribocytidilic acid (PolyI:C) are used to induce maternal immune activation (MIA), resulting in offspring developing a SCZ-like phenotype. This model has been described extensively [11], with SCZ-like alterations observable in behavior [12] and neurobiology [13,14], as well as peripheral changes, e.g., in the microbiome [15].

Previous studies using this model have identified high-frequency DBS in the medial prefrontal cortex (mPFC) as one of the most promising protocols [16]. In adult animals, this protocol has been shown to improve two hallmark behavioral impairments tested in animal models for SCZ: sensorimotor gating and latent inhibition [17]. When applied during adolescence, DBS in the mPFC can prevent impairments in sensorimotor gating and latent inhibition in adulthood, while counteracting alterations in brain structure and neurotransmitter systems [18]. Additionally, alterations in microglia, a key aspect of neuroinflammation that accompanies and possibly underlies symptoms of SCZ [19], are partially normalized [20].

Prior work employing electrophysiological recordings in key areas of the limbic system has shown that DBS in the mPFC partly improves MIA-induced impairments in theta and gamma synchronicity. Additionally, increased gamma/theta cross-frequency coupling was partly reduced [21]. In the present study, we utilized a similar protocol to further explore effects in the limbic system. Through an exploratory approach in a small sample, we aimed to better understand the time course of the effects of DBS, and assess alterations in single-unit observations.

## 2. Materials and Methods

### 2.1. Animals

Pregnant Wistar rats (Charles River, Sulzfeld, Germany) were obtained at gestational day (GD) 13 and received intravenous injections of 4 mg/kg PolyI:C (Sigma-Aldrich, Taufkirchen, Germany, P1530) or 1 mL/kg vehicle (0.9% NaCl) at GD 15. Pups were weaned at postnatal day 21 and housed in groups of four animals of the same sex. Only male rats, one per litter, were used for the experiments. The experimenter was blinded to the condition of the animal (i.e., treatment of the mother). Animals were housed with food and water ad libitum under a 12 h:12 h light/dark cycle (lights on at 6 am). In total, 16 male rats, 7 rats in the MIA model and 9 control rats, were used at an age of around 90 days. All experiments were carried out in agreement with the principles regarding the care and use of animals adopted by the German Animal Welfare Law after approval by the *LANUV* (Landesamt für Natur, Umwelt und Verbraucherschutz Northrhine-Westfalia, 84-02.04.2011.A299).

### 2.2. Deep Brain Stimulation and Electrophysiology

DBS and electrophysiology recordings were performed as previously described [21]. In short, animals received long-term anesthesia via injections of urethane (1.6 g/kg, i.p.) and ketamine (0.06 g/kg, i.p.) and were placed in a stereotaxic frame. Urethane was chosen as the principal anesthetic because it provides a stable, long-lasting plane of anesthesia while largely preserving cortical and hippocampal oscillatory activity. Ketamine was added to provide analgesia during induction and surgery, permitting the urethane dose to be kept at the lower end of the effective range. For recording, glass-isolated tungsten electrodes (ca. 1–2 MOhm) were placed into the mPFC (AP: 2.7 mm, ML: −1 mm, DV: −3.55 mm), the nucleus accumbens (NAc) (AP: 1.2 mm, ML: −2.0 mm, DV: −7.0 mm), the ventral hippocampus (vHC) (AP: −5.6 mm, ML: −4.5 mm, DV: −7.0 mm), and the ventral tegmental area (VTA) (AP: −6.0 mm, ML: −0.7 mm, DV: −8.0 mm) of the left hemisphere, according to Paxinos & Watson [22] (1998 DBS was performed using a concentric bipolar stimulation electrode (center 0.75 × 0.1 mm, outer ring 0.25 × 0.25 mm, Lohmann Research equipment, Dortmund, Germany) within the prelimbic area of the mPFC, around 1 mm rostral of the recording electrode. Stimulation lasted for 45 min, with a high-frequency protocol at 130 Hz using 150 µA biphasic pulses with 90 µs pulse duration [18]. Activity was recorded for 10 min before, as well as 10 min after the DBS protocol.

### 2.3. Data Analysis

For the analysis of baseline differences, the 10 min recording period preceding the DBS protocol was compared between groups. Acute DBS effects were assessed during the first 60 s following DBS, while the subsequent 9 min were used to measure longer-term effects. Local field potentials (LFP) from all recording sites were analyzed. LFP data from 3 animals (two MIA and one control) was lost due to technical problems. Band power contribution to overall spectral power was normalized as a percentage of total power in order to account for different recording times. Principal component cluster analysis integrated in Spike 2 (Cambridge Electronic Design, Cambridge, UK) was used to identify 2 distinct neurons in nearly all recording sites (with just one neuron in the NAc in one control and one MIA animal). Spikes and bursts of spikes, beginning with a spike interval of ≤5 ms and a maximal spike-interval of 50 ms, were analyzed.

For LFPs, a repeated measures ANOVA with time as a within-subject factor and group (MIA vs. control) as a between-subject factor was performed. For the analysis of single-unit data, a mixed-effects model with time and group as fixed effects and animal as a random effect was used. For each observation and time condition, outliers were identified using Grubbs’ test. An animal identified as an outlier in any time window was excluded from that measure across all three time windows. Kenward–Roger approximation was used for degrees of freedom, and effect sizes are reported as η^2^. Post hoc comparisons were conducted using Bonferroni-corrected *t*-tests. Data analysis was performed using R (v.4.1.3 [23]), using the packages “psych” (v. 2.4.3 [24]), “dplyr” (v.1.1.2 [25]) and “ggplot2” (v. 3.4.4 [26]). The threshold for statistical significance was set at *p* ≤ 0.05. Descriptive statistics for all analyzed data can be found as Supplementary Material (Table S1: LFPs, Table S2: single-unit data).

## 3. Results

### 3.1. Group Differences

Significant main effects of group were observed for LFPs in the mPFC and spikes in the vHC. Relative alpha-band power in the mPFC (F(1, 10) = 10.39, *p* = 0.009, η^2^ = 0.51, 95%-CI: [0.12, 1.00]; Figure 1A) was significantly decreased in MIA rats (0.044 (SE = 0.021, 95%-CI: [−0.002, 0.090])) compared to controls (0.126 (SE = 0.015, 95%-CI: [0.093, 0.158])). Relative beta-band power in the mPFC (F(1, 9) = 33.70, *p* < 0.001, η^2^ = 0.79, 95%-CI: [0.49, 1.00]; Figure 1B) was also lower in MIA offspring (0.018 (SE = 0.012, 95%-CI: [−0.009, 0.045])) compared to controls (0.106 (SE = 0.009, 95%-CI: [0.085, 0.127])).

Additionally, MIA offspring showed overall lower spikes per minute in the vHC (F(1, 28) = 4.73, *p* = 0.028, η^2^ = 0.14, 95%-CI: [0.00, 1.00]; Figure 2E), with lower spikes in MIA animals (269 (SE = 157, 95%-CI: [−52.7, 591])), compared to controls (723 (SE = 137, 95%-CI: [441.5, 1004])). All other analyzed LFPs and single-unit recordings did not show a significant main effect of group.

### 3.2. Effects Following Deep Brain Stimulation

Following the DBS protocol, the effects of stimulation become apparent in LFPs, as well as in single-unit recordings. In the NAc, a main effect of time was observed for relative gamma-band power (F(2, 18) = 6.02, *p* = 0.010, η^2^ = 0.40, 95%-CI: [0.08, 1.00]; Figure 1C). Here, relative gamma-band power was significantly higher 60 s post stimulation (0.088 (SE = 0.015, 95%-CI: [0.057, 0.116])), compared to the time before stimulation (*p* = 0.012; 0.025 (SE = 0.015, 95%-CI: [−0.006, 0.056])) and the remaining time post stimulation (*p* = 0.014; 0.026 (SE = 0.015, 95%-CI: [−0.005, 0.056])). For relative theta-band power in the NAc, a significant interaction was observed (F(2, 20) = 4.96, *p* = 0.018, η^2^ = 0.33, 95%-CI: [0.04, 1.00]; Figure 1D). In the first 60 s following stimulation a significant difference (*p* = 0.005) became apparent, with MIA rats (0.133 (SE = 0.023, 95%-CI: [0.087, 0.180])) showing significantly reduced relative theta-band power compared to controls (0.197 (SE = 0.016, 95%-CI: [0.164, 0.230])).

A main effect of time (F(2, 18) = 7.11, *p* = 0.005, η^2^ = 0.44, 95%-CI: [0.12, 1.00]; Figure 1E), as well as an interaction effect (F(2, 18) = 4.87, *p* = 0.02, η^2^ = 0.35, 95%-CI: [0.04, 1.00]) could also be observed for relative gamma-band power in the vHC, not surviving post hoc testing.

Single-unit recordings showed an additional interaction effect on spikes per minute in the vHC (F(2, 56) = 3.83, *p* = 0.028, η^2^ = 0.12, 95%-CI: [0.01, 1.00]; Figure 2E). At 60 s post stimulation, MIA rats (707.4 (SE = 249, 95%-CI: [212, 1203])) showed significantly fewer spikes per minute than controls (*p* < 0.001, 1870.5 (SE = 218, 95%-CI: [1437, 2304])).

For all recording sites a main effect of time was observed, with spikes and bursts per minute being significantly higher 60 s following stimulation compared to the other time points (Figure 2A–H, see Table S2 for statistical parameters).

## 4. Discussion

The aim of the present study was to gain insights into possible mechanisms of DBS for SCZ by further investigating electrophysiological correlates in the PolyI:C rat model. Offspring following MIA showed overall decreased alpha- and beta-band power in the mPFC. In the NAc, MIA offspring showed a significantly decreased theta-band power 60 s past stimulation, while gamma-band power was significantly increased in both groups.

Single-unit recordings at all recording sites showed transient responses to DBS, with increased numbers of spikes and bursts 60 s after stimulation. On the single-unit level, just the number of spikes in the vHC showed a group difference, with an overall lower number of spikes in MIA rats. Previous work has reported reduced firing rates and reduced population activity of VTA dopamine neurons in adult male MIA offspring [27], but this effect could not be reproduced in this study.

Reduced alpha [28]- and beta-band power in SCZ patients have been reported, with alpha-band power showing correlations with symptom severity and chronicity [29]. Disruptions in beta band power have primarily been associated with negative symptoms [30]. The reduced alpha- and beta-band power observed in the mPFC of MIA offspring is consistent with these findings. This aligns with previous reports of altered hippocampal firing following MIA, where firing rates were reduced during navigation [31], suggesting that MIA does not produce a uniform change in excitability but rather disrupts firing patterns in a region-specific manner.

Stimulation of the mPFC was followed by a transient response that was not restricted to a single target region. At all four recording sites, the number of spikes and bursts increased within the first 60 s after stimulation and did not significantly differ from baseline thereafter. In parallel, gamma-band power in the NAc increased in the same time window in both groups. These effects represent a general response to prefrontal stimulation rather than a model-specific effect. Two group-specific responses to stimulation were observed, both confined to the first minute after stimulation and both in the same direction. Relative theta-band power in the NAc did not differ between groups at baseline, but MIA animals showed significantly lower values than controls 60 s after stimulation, with no difference over the subsequent nine minutes. In the vHC, a corresponding interaction was found for firing rate: MIA animals showed markedly fewer spikes per minute than controls in the same 60 s window. Taken together, these findings indicate that the general response to prefrontal stimulation described above is attenuated in MIA offspring in two downstream target regions, detected by two measures. Given that dysfunctional theta-band dynamics impair cognitive control in patients with SCZ, and that non-invasive prefrontal stimulation can temporarily improve adaptive control in these patients [32], an attenuated acute response to prefrontal stimulation in MIA offspring may be relevant to the limited efficacy of single stimulation sessions. Knowledge of accumbal theta activity in MIA models remains limited, although theta sequences have been reported to be disrupted in this model more generally [31].

It is important to consider some limitations when interpreting these results. The nature of the study was exploratory and results have to be considered in light of the small sample size. The statistical approach is very susceptible to producing false positive results, making it necessary to replicate findings in a larger scale study. In addition, the present study only used male animals. It has previously been shown that the effects of MIA can be sex dependent [12], highlighting the necessity to also include female animals in future studies. With the current approach, results cannot be generalized for both sexes.

The anesthetic regime warrants particular consideration here, since ketamine is an NMDA receptor antagonist and is itself used as a pharmacological model of schizophrenia [33]. Both groups received an identical regime, so anesthesia by itself is unlikely to generate the group differences reported here. It can, however, not be ruled out that MIA and control animals differ in their sensitivity to NMDA receptor antagonism, and such an interaction would be difficult to distinguish from a model effect in the present design. Additionally, urethane and ketamine anesthesia are both known to influence oscillations and alter brain states [34,35]. This may affect baseline levels and responsiveness to DBS. The two anesthetics are also expected to affect the measured frequency bands differentially. Residual ketamine elevates gamma-band power [36], which is relevant to the interpretation of absolute gamma values. Urethane, by contrast, produces spontaneous alternation between slow-wave and theta-dominated states on a timescale of minutes [37]. While anesthesia enables stable recordings, these conditions may not fully reflect the dynamics in awake animals, and replication in awake animals, or under an anesthetic without direct glutamatergic action, remains necessary before these oscillatory signatures can be regarded as independent of the anesthetic state. As there was no sham group included for the exploratory approach, differences following DBS could be a result of time under anesthesia.

Furthermore, stimulation and recordings were confined to the left hemisphere in all animals. This unilateral approach takes into account the organization of the circuit under investigation with mostly ipsilateral projections. The left hemisphere was used throughout, consistent with the stimulation protocol that has been established in the lab. Keeping the side constant across animals also removes hemisphere as a source of between-animal variance. However, potential effects of lateralization should not be disregarded [38], as they appear to play a significant role in SCZ [39]. This is especially important because we cannot establish whether the MIA-associated group differences reported here are themselves lateralized, nor whether comparable effects would be obtained in the right hemisphere. This is particularly relevant in this model, as MIA has been shown to alter behavioral asymmetry [14], and lateralization is increasingly recognized as an underappreciated source of variance in rodent neuroscience [38]. Bilateral recording combined with unilateral stimulation would be the most informative next step. Beyond addressing the question of lateralization, it would allow the unstimulated hemisphere to serve as a within-animal control for the time-dependent changes reported here, which the present design cannot separate from stimulation-independent drift during the recording session.

## 5. Conclusions

Taken together, this study revealed that MIA in the PolyI:C rat model induces significant electrophysiological alterations in offspring, including reduced alpha- and beta-band power in the mPFC and decreased neuronal spiking in the vHC. These findings parallel known electrophysiological abnormalities in SCZ patients and suggest potential mechanisms underlying cognitive and behavioral deficits. Effects following the DBS protocol could be observed, particularly highlighting a role for theta power in the NAc. These results provide further evidence for the potential effects of stimulation of the mPFC on the limbic system. The approach should be further explored in larger scale animal studies in order to shed light on the mechanisms of deep brain stimulation and, ultimately, inform future directions in psychiatric care.

## Figures and Tables

**Figure 1:**
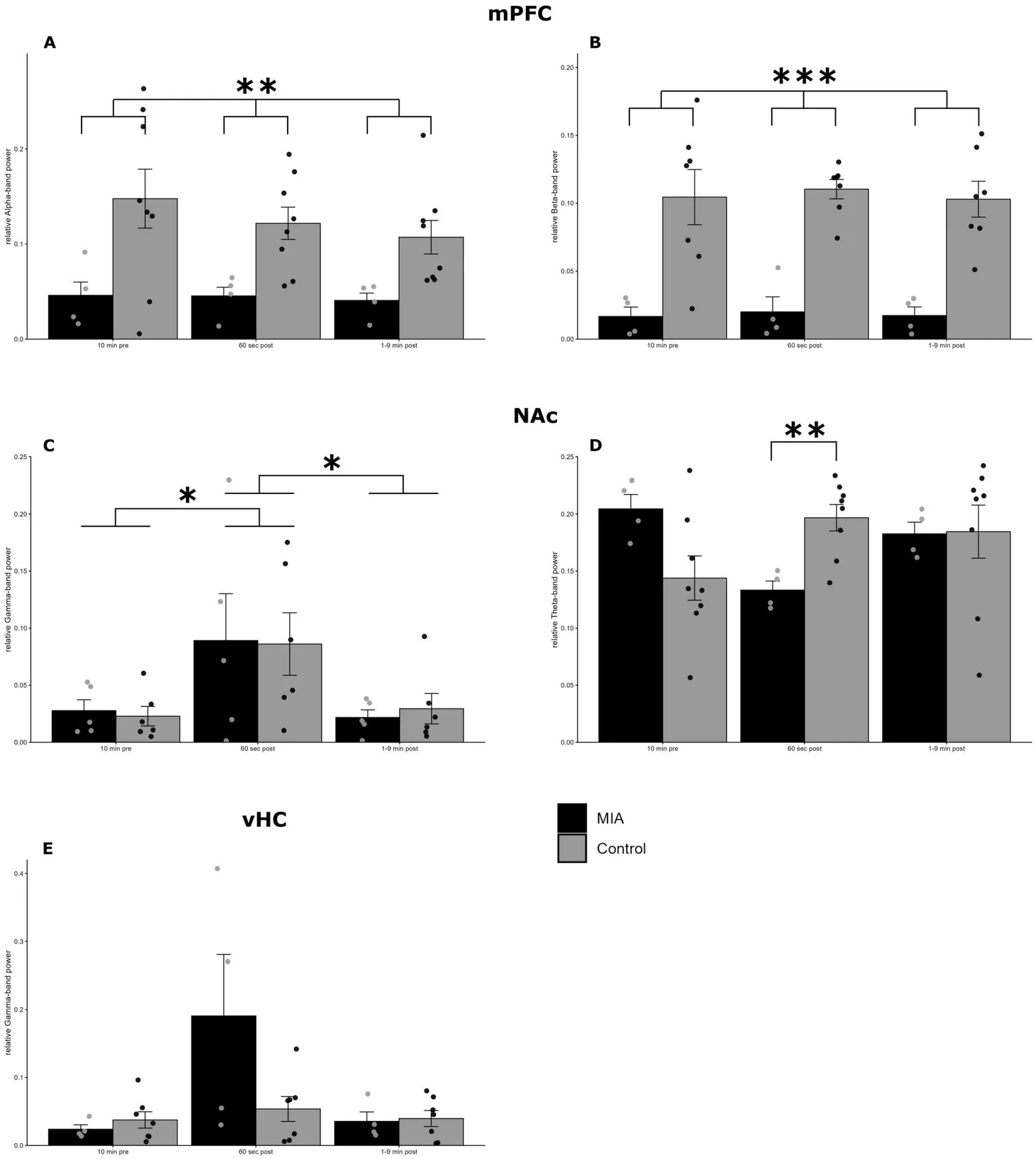
Local field potentials in maternal immune activation (MIA) and control animals before (10 min pre) and after (60 s post, 1–9 min post) deep brain stimulation. MIA decreased alpha- (**A**) and beta-band (**B**) power in the medial prefrontal cortex (mPFC). In the nucleus accumbens (NAc), gamma-band power increased immediately after stimulation, compared to the other time points (**C**) and theta-band power decreased in MIA rats directly following stimulation (**D**). Effects on relative gamma-band power in the ventral hippocampus (vHC) (**E**) did not survive post hoc testing. Error bars indicate standard error of the mean; * *p* ≤ 0.05, ** *p* ≤ 0.01, *** *p* ≤ 0.001.

**Figure 2:**
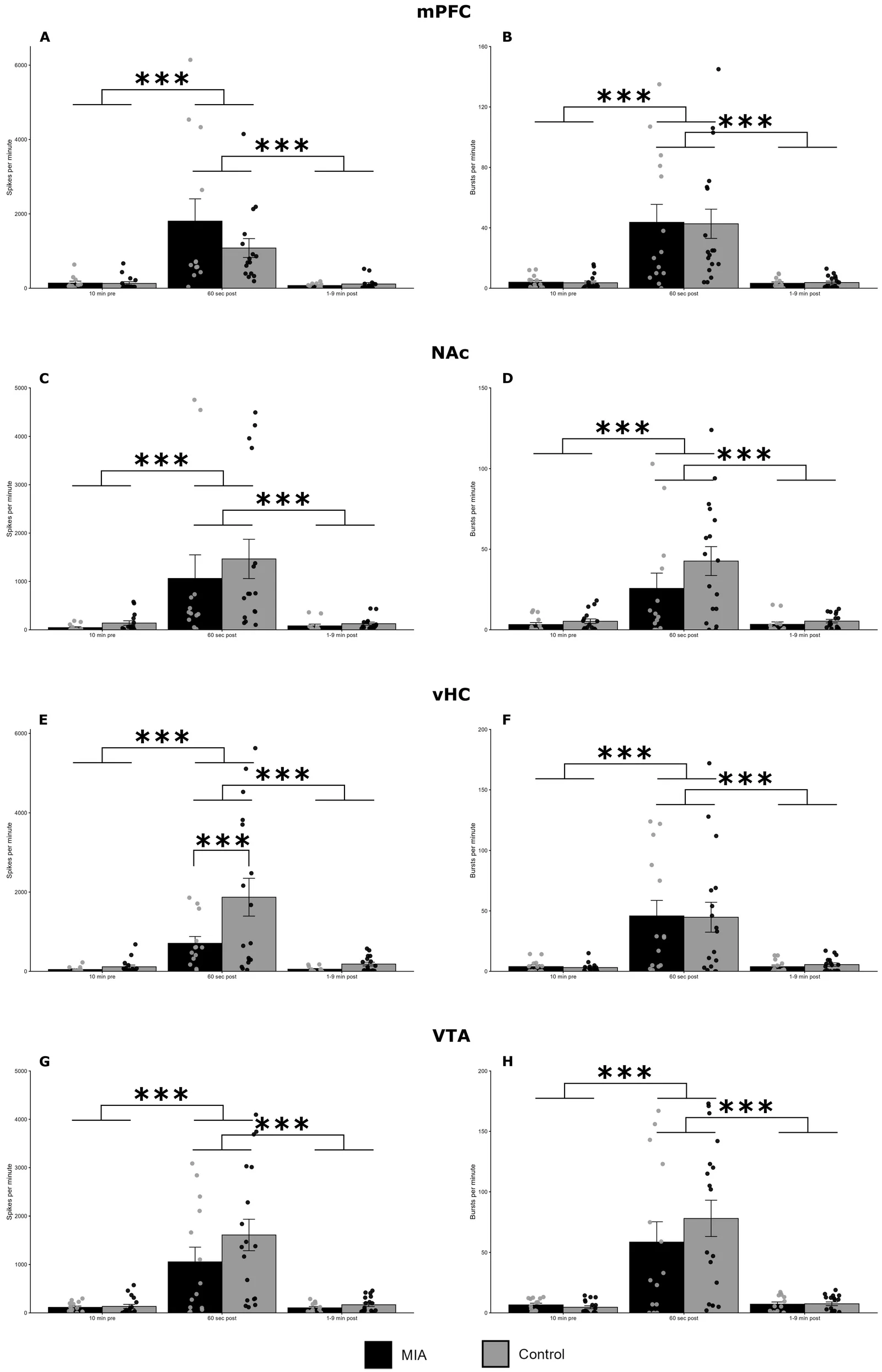
Single-unit recordings in maternal immune activation (MIA) and control animals before (10 min pre) and after (60 s post, 1–9 min post) deep brain stimulation. In the medial prefrontal cortex (mPFC), the nucleus accumbens (NAc), the ventral hippocampus (vHC) and the ventral tegmental area (VTA) the number of spikes and bursts per minute were significantly increased 60 s after stimulation compared to the other time points (**A**–**H**). In the vHC there is a significant difference at 60 s post stimulation, with MIA rats showing fewer spikes per minute (**E**). Error bars indicate standard error of the mean; *** *p* ≤ 0.001.

## Data Availability

The raw data supporting the conclusions of this article will be made available by the authors on request.

## Supplementary Materials

The following supporting information can be downloaded at https://www.mdpi.com/article/10.3390/brainsci16101003/s1, Table S1: LFPs; Table S2: single-unit data.
